# Social Touch, Social Isolation, and Loneliness in Borderline Personality Disorder During the COVID-19 Pandemic

**DOI:** 10.3389/fpsyt.2022.876413

**Published:** 2022-06-23

**Authors:** Anna Schulze, Miriam Biermann, Konstantina Atanasova, Franziska Unterseher, Louisa Winkler, Martin Bohus, Stefanie Lis

**Affiliations:** ^1^Department of Clinical Psychology, Central Institute of Mental Health, Medical Faculty Mannheim, University of Heidelberg, Heidelberg, Germany; ^2^Department of Psychosomatic Medicine and Psychotherapy, Central Institute of Mental Health, Medical Faculty Mannheim, University of Heidelberg, Heidelberg, Germany

**Keywords:** social touch, loneliness, social distancing, Borderline Personality Disorder, COVID-19 pandemic

## Abstract

**Background:**

Interpersonal impairments in borderline personality disorder (BPD) are characterised by a lack in the sense of belonging and the fear of being excluded. One feature of interactions that can promote a sense of social belonging is interpersonal touch. While some studies suggest that individuals with BPD experience social touch as less pleasurable than healthy individuals (HCs), there are no studies that investigated whether this difference is associated with feeling less socially connected. This question is particularly important during the COVID-19 pandemic, since one central behavioural recommendation is “social distancing”. An increase in loneliness has been discussed as a consequence and it has been suggested that individuals with BPD may be particularly burdened. However, the primary goal of “social distancing” is not preventing social contacts, but physical proximity. In our study we investigated the interplay between feeling close to others, contact frequency and the appraisal of social touch in BPD. We were additionally interested in whether these factors contribute to the burden through “physical distancing”.

**Methods:**

We assessed subjective and objective social isolation, the need, importance, and liking of social touch, as well as the burden through “physical distancing” policies in 130 women (61 BPD and 69 HCs).

**Results:**

Participants of the BPD group reported higher loneliness, less social contacts and a lower need for, importance and liking of social touch compared to HCs. Larger social networks, higher frequency of in-person contacts and higher liking and importance of social touch were associated with lower levels of loneliness. Both groups did not differ regarding their burden through “physical distancing”. A higher need for and lower importance of social touch predicted a higher burden through “physical distancing”.

**Conclusions:**

A positive appraisal of social touch was associated with less loneliness, independently of an individual's objective social isolation. In BPD, impairments of this fundamental facet of social interaction might hamper forming and strengthening of social bonds and contribute to the patients' interpersonal dysfunction. Changing the attitude towards social touch and in consequence its liking and importance in social interaction might provide one avenue to improve the sense of social connectedness in these patients.

## Introduction

“Social distancing” is one of the main behavioural restrictions aiming to reduce the spread of the COVID-19 infections during the COVID-19 pandemic. The associated changes in every-day life may challenge one of the fundamental needs of human beings, that is, the need for social belonging (1). While this imposes a burden on most people, it has been suggested that this burden might be higher in individuals with a mental disorder (2–4). Individuals with Borderline Personality Disorder (BPD) may be particularly affected: The threat to being socially connected imposed by “social distancing” might intensify the fear of being abandoned and the feeling of being disconnected from others, which are characteristic of BPD (5–9). Moreover, “social distancing” might also reduce the size and diversity of an individual's social network, potentially having a disproportionally strong effect on loneliness in BPD patients, who already have smaller and less diverse social networks (6, 7, 10, 11). In a first investigation of the impact of the COVID-19 pandemic and its mandated restrictions in a small sample of patients with mood disorders, especially individuals with BPD (*n* = 16) reported a deleterious impact on their symptomatology (12).

During the COVID-19 pandemic, especially when cases were peaking, governments recommend “social distancing” and even enforced these behavioural restrictions by mandated “lockdowns”. However, as the American Centres for Disease Control and Prevention state on their website “*social distancing, …also called “physical distancing,” means keeping a safe space between yourself and other people who are not from your household*” (13). Thus, the primary goal of “physical distancing” is not to prevent social contacts, but rather physical proximity in order to limit the spreading of the infection.

In line, one of the challenges people are faced with during the COVID-19 pandemic is to preserve social contacts and prevent the feeling of being lonely, while simultaneously reducing physical proximity. Beyond more traditional approaches such as writing letters or using phones, digital communication technologies like social media platforms and video calls allow people to stay in touch without meeting in person. The use of these technologies has already increased continuously before the pandemic (14). However, in non-pandemic times the use of virtual technologies has also been linked to higher levels of loneliness (15, 16). Hence, it is still up for debate whether their use is the cause or the consequence of feeling lonely, that is, being less socially connected than one would wish (17, 18). Moreover, their use does not seem to be particularly effective in counteracting loneliness, at least in the elderly (19).

So far, empirical data on the use of virtual technologies in individuals with BPD are missing. A recent study by Ooi, Michael (20) revealed that people high in BPD features ascribe a high importance to social media in their daily life. However, since communication *via* virtual channels restricts, at least partially, the availability of social cues such as body language, it might leave more room for ambiguity during social contacts. Since changes in social cognitive processing of ambiguous social cues are one feature of interpersonal impairments in BPD (21–24), it is questionable whether individuals with BPD use virtual communication channels during “physical distancing” policies to a comparable extent as healthy individuals and whether this is comparably helpful in reducing the experience of loneliness during the COVID-19 pandemic. On the other hand, social media offers people the possibility to present themselves online in a way they want to be seen. This could be helpful for people with BPD, as shame experience is elevated in this group (25). Thus, it is also conceivable that people with BPD use virtual communication channels as much as or even more often than healthy individuals.

Social contacts by virtual technologies reduce physical proximity during social encounters as intended by physical distancing policies. However, they also prevent social touch like hugging one another or linking arms, which is an important component of social interactions. Social touch might even promote prosocial behaviour among strangers; waiters, for example, receive greater tips when they touch customers (26). Receiving affectionate touch by someone close is positively associated with physical, social and psychological wellbeing in children, adolescents and adults (27–29). Moreover, social touch promotes a sense of security, reduces subjective pain, and facilitates stress regulation (30–33). In line with these findings, social touch has been shown to reduce feelings of loneliness (34). “Touch-hunger”, an increased need for social touch, has been discussed as a consequence of “physical distancing” policies (35, 36).

Despite touch being an important component of interpersonal encounters in general, the preference for physical proximity differs between people (37, 38). For example, individuals with BPD prefer a larger interpersonal distance (39–41). One reason might be the high prevalence of adverse childhood experiences (ACE) in BPD (42, 43). Individuals with a history of ACE and a lack of caring touch in childhood are less sensitive to affective touch, reveal an attenuated neuronal response to affective touch, and prefer a greater interpersonal distance to strangers (44–46). Particularly, ACE associated with physical neglect and abuse have been proposed as essential for the interpretation of touch as intrusive and less pleasant (47, 48).

Attachment styles developed in childhood are associated with different long-term effects following adversity in childhood (49–52). Adults with insecure attachment styles experience gentle touch as less pleasant (53), report less positive feelings towards affectionate touch in romantic relationships (47, 48), show less comfort with close contact in interaction with their children (54), and discriminate less between affective and non-affective touch (55). Beyond these relationships between attachment style and alterations in the processing of social touch, an insecure attachment style is also linked to higher levels in the experience of loneliness (56, 57). Both, BPD and ACE have been associated with insecure attachment in adulthood (58–61). In line with the aforementioned, individuals with BPD perceive social touch as less pleasant compared to healthy individuals (62). This suggests that the lack of physical proximity during “physical distancing” affects these individuals to a lower extent. However, these findings do not necessarily imply that the affected individuals also experience a lower need for physical closeness and that touch is less important during social contacts. So far, empirical studies on these different facets of social touch in BPD and ACE are missing as well as studies on their potentially differential impact on the experience of loneliness and the burden associated with “physical distancing” during the COVID-19 pandemic.

For many months the discussion about the consequences of “physical distancing” on mental health focussed on whether the reduction of in-person contacts result in an increase of loneliness and whether virtual communication channels can counteract this development. However, the extent to which “physical distancing” during the COVID-19 pandemic led to feelings of loneliness might not only be influenced by contact frequencies and channels, but also by the appraisal of physical proximity during social encounters. However, there is a lack of studies investigating whether the appraisal of interpersonal touch modulates the relationship between in-person contacts and virtual contacts with the feeling of being socially disconnected.

The present study aims to contribute to the understanding of the interplay between objective and subjectively experienced social isolation, the appraisal of social touch in social contacts, and the use of different communication channels in healthy individuals and individuals with BPD. Moreover, we were interested in whether a history of ACE affects the appraisal of interpersonal touch in BPD and whether an individual's attachment style modulates this relationship. We expected (1) that individuals with a history of BPD report smaller and less diverse social networks, a higher level of subjective social isolation and that they appraise social touch as less pleasant compared with healthy controls. Moreover, we hypothesised that (2) the frequency of social contacts partially predicts the feeling of social isolation and that (3) the appraisal of social touch moderates this relationship, depending on the used communication channels. In more detail, we expected that in participants who appraise social touch as more pleasant, the relationship of in-person contacts to feelings of social isolation is stronger and for the association of virtual contacts to feelings of social isolation attenuated. To further investigate the factors underlying inter-individual differences in the appraisal of social touch during social contacts, we analysed its relationship with both ACE and attachment style. We expected that (4) social touch is appraised as less pleasant in those with a history of ACE and that this association is particularly strong in those with a lower ability to feel close towards others, as one dimension of attachment. Beyond these research questions addressing interpersonal functioning independently of the COVID-19 pandemic, we additionally explored whether these facets of social interaction are related to the burden through “physical distancing” due to a lockdown during the COVID-19 pandemic. In line with previous findings indicating preferences for a higher interpersonal distance and less pleasant appraisal of social touch in BPD patients, we expected that those individuals who appraise interpersonal touch as less pleasant, feel less burdened by physical distancing.

## Materials and Methods

An online survey was conducted between February 13 and April 4, 2021. During this period, Germany had strict restrictions (“lockdown”) in place. Most public facilities were closed, face masks were mandatory on public transport, in supermarkets, and in places where it was not possible to maintain a 1.5 m distance to others. Regarding private gatherings, meetings were only allowed between one household and one extra person.

### Participants

Participants were recruited from the database of the central project of the KFO 256, a Clinical Research Unit funded by the German Research Foundation dedicated to investigating mechanisms of disturbed emotion processing in BPD (63). The analyses in the present paper are based on survey data of 130 women of which 69 were healthy controls and 61 individuals who had met the *Diagnostics and Statistical Manual of Mental Disorders* (DSM-IV) (64) diagnosis of BPD in the past, that is, met at least five of the nine *DSM–IV* criteria for BPD, as assessed by trained clinical psychologists using the International Personality Disorder Examination (IPDE) (65). For further details on the recruitment procedure, see Supplementary Material A. All of these individuals gave informed consent before participating in the survey. The study was approved by the Research Ethics Board II of the Medical Faculty Mannheim of Heidelberg University.

Sociodemographic features, BPD psychopathology and severity of childhood trauma for both groups are reported in Table 1. We measured the BPD symptom severity with the short version of the Borderline Symptom List (BSL-23) (66), the level of BPD features with the Borderline Scale from the Personality Assessment Inventory (PAI-BOR) (67), German version VEI-BOR (68); and severity of depressive symptoms with the Beck Depression Inventory II (BDI-II) (69), German version (70); range: 0–63; Cronbach's α in the current study were α = 0.96 for the total sample, α = 0.94 in the BPD and α = 0.87 in HC sample). The severity of childhood trauma was based on self-reports measured with the short form of the Childhood Trauma Questionnaire (CTQ-SF) (71), German version (72). For further details, see Supplementary Materials A, B.

**Table 1:** Sample characteristics.

	**HC (*****N*** **=** **69)**		**BPD (*****N*** **=** **61)**				***P*-value**
	***M***	***SD***	***M***	***SD***			
Age	33.22	7.23	33.62	8.86	*t* = −0.29		0.775
**Family status**
Single	23.19%		36.07%		χ^2^ (1) = 2.60		0.107
Relationship	76.81%		63.93%				
**Education**
Low	0.00%		1.64%		Fisher's exact test, two-sided		0.459
Intermediate	13.04%		19.67%				
High	81.16%		75.41%				
Other	5.80%		3.28%				
**Living situation**
Alone	26.09%		44.26%		χ^2^ (1) = 4.73		0.030
With others	73.91%		55.74%				
**Occupation**
Full time	55.07%		26.23%		χ^2^ (1) = 11.09		0.001
Other	44.93%		73.77%				
**Current treatment**
Yes	1.45%		72.13%		χ^2^ (1) = 71.47		<0.001
No	98.55%		27.87%				
**Psychopathology and borderline features**
	**HC**		**BPD**		***t***	***P*** **-value**	***d***
	***M***	***SD***	***M***	***SD***			
BSL23	0.27	0.29	1.97	0.80	−15.62	<0.001	−2.88
VEI-BOR	16.80	8.08	50.54	6.30	−26.30	<0.001	−4.62
BDI-II	7.54	5.92	28.98	12.12	−12.56	<0.001	−2.29
CTQ total score	28.62	3.27	59.89	19.70	−12.99	<0.001	−2.28
Emotional abuse	6.16	1.57	16.52	5.62	−13.93	<0.001	−2.58
Physical abuse	5.16	0.53	7.44	4.03	−4.39	<0.001	−0.82
Sexual abuse	5.06	0.29	8.57	6.33	−4.33	<0.001	−0.81
Emotional neglect	6.72	1.86	17.36	5.54	−14.29	<0.001	−2.64
Physical neglect	5.52	1.16	9.98	4.10	−8.21	<0.001	−1.52

Both groups were balanced for age, education and relationship status (all *ps* > 0.1). However, individuals of the BPD group were more likely to live by themselves [χ^2^ (1) = 4.73, *P* = 0.030, ϕ = 0.19], worked less often full-time [χ^2^ (1) = 11.09, *P* = 0.001, ϕ = 0.29] and currently received psychotherapeutic, psychopharmacologic and/or psychiatric treatment to a higher percentage [χ^2^ (1) = 71.47, *P* < 0.001, ϕ = 0.74]. The BPD group reported a higher level of BPD symptoms (BSL-23), BPD features (VEI-BOR), depressive symptoms (BDI-II) and a higher severity of ACE (CTQ) compared with the HC group (see Table 1). According to the severity categories proposed by Kleindienst, Jungkunz (73), the mean BSL-23 scores obtained in our sample indicate a high level of BPD symptoms in the BPD group and none to low BPD symptoms in the HC group.

### Measures

#### Loneliness

Loneliness, that is, the subjective experience of social isolation, was assessed using the Revised University of California Los Angeles Loneliness Scale (ULS-R) (74), German version (75).

The ULS-R consists of 20 items. Following the recommendations of the authors of the German validation study (75), items were rated on a 5-point Likert scale, instead of a 4-point Likert scale, ranging from 1 (*not true at all*) to 5 (*completely true*). Items were combined in a sum score (range 20–100) with higher scores indicating higher levels of loneliness. Internal consistency for the ULS-R was α = 0.96 (BPD: Cronbach's α = 0.91; HCs: Cronbach's α = 0.90).

To capture the subjective experience of social isolation in relation to an individual's actual social network, we additionally measured the feeling of closeness in the context of the social network index (see below).

#### Social Touch

We assessed three facets of the appraisal of social touch in social relationships: (a) an individual's general attitude towards interpersonal touch with a self-report questionnaire, (b) the liking of social touch in an experimental task, as well as (c) the importance of different kinds of touch towards the members of an individual's social network.

The need for interpersonal touch was assessed by the Need for Interpersonal Touch Scale (NFIPT) (76). The 20-item NFIPT measures an individual's general attitude towards interpersonal touch on a 7-point Likert scale ranging from 1 (*not at all true*) to 7 (*exactly true*), combined in a mean score (range 1–7) with higher scores indicating a higher need for interpersonal touch. In the present study internal consistency for the NFIPT Scale was α = 0.93 (BPD: Cronbach's α = 0.93; HCs: Cronbach's α = 0.91).

We assessed an individual's liking of social touch with an experimental task during which participants watched video-clips of socio-affective touch sequences and rated how pleasant they would experience the touch displayed on a 7-point-Likert scale ranging from −3 (*very unpleasant*) to +3 (*very pleasant*). Independent variables were the valence of the displayed touch (negative touch e.g., shaking or slapping; positive touch e.g., hugging or stroking) and the social context (non-social: touching an object; social: touching another person). Participants rated 60 video clips overall (15 clips for each of the experimental conditions of this 2 × 2 design). Video clips were selected from the socio-affective touch data base (77), Stimuli selected: positive: 2–6, 15–19, 28–32 (social), 40–44, 52–56, 64–69 (non-social), negative: 8,10–13, 21, 23–26, 34, 36–39 (social), 46,48–51, 58,60–63,70, 72–75 (non-social). The video clips were presented in six blocks. The sequence of the blocks as well as the videos within each block were presented in random order.

To measure the appraisal of interpersonal touch within an individual's actual social network, we additionally asked participants to judge the importance of interpersonal touch in the context of the social network index (see below).

#### Social Network and Use of Communication Channels

We measured participants' objective social isolation with the Social Network Index (SNI) (78). Based on 12 different social domains, the SNI quantifies the size of the network, that is, the number of people with whom an individual speaks at least once every 2 weeks (SNI-size), and the diversity of the network, that is, the number of social domains in which the respondent has regular contact with at least one person (SNI-diversity).

Since social touch is restricted to social contacts during which people meet in person, we additionally asked participants to assess in each of the social domains how often they used different communication channels for social contacts during the last 4 weeks of the lockdown. Beyond in-person contacts we asked how often participants used different digital communication channels (for further details see Supplementary Material C). Participants estimated the frequency of contacts on a 7-point Likert-scale, ranging from 1 (*not at all*) to 7 (*several times a day*).

We additionally asked participants to rate the closeness and the importance of interpersonal touch towards others for each of the 12 social domains on a 7-point-Likert scale (range: 1–7, closeness: *not at all* to *very close*; importance of touch*: not important at all* to *very important*). Ratings of closeness (“How close to do you feel towards these persons?”) were averaged across the 12 social domains for further analyses. Participants rated the importance of touch for six different types of social encounters (adapted from) (79). For further details, see Supplementary Material D. Ratings of the importance of touch were averaged across the 12 social domains and the six social situations for further analyses.

#### Attachment

Attachment-related attitudes were assessed with the Adult Attachment Scale (AAS) (80), German version (81). The scale consists of 15 items, assessing the three subscales closeness (needing intimacy and being comfortable with it; BPD: Cronbach's α = 0.84; HC: Cronbach's α = 0.80), dependence (trust that others are available when needed; BPD: Cronbach's α = 0.76; HC: Cronbach's α = 0.76) and anxiety (fear of not being loved and abandoned; BPD: Cronbach's α = 0.61; HC: Cronbach's α = 0.56), thus measuring different dimensions of attachment. Participants were asked to indicate their level of agreement with these 15 statements on a 5-point Likert scale ranging from 1 (*not true at all*) to 5 (*completely right*). Subscale scores range from 5 to 25.

#### Burden Through Physical Distancing Measures

Participants assessed how strongly they felt burdened by different physical distancing measures. Items were adapted from the Covid-19 Snapshot Monitoring Survey (82). All questions were rated on a 6-point Likert-Scale ranging from 1 (*not at all*) to 6 (*very*). Since the perceived burden might be influenced by compliance to physical distancing measures, we additionally asked participants to judge how strongly they complied with the different behavioural recommendations. For further details, see Supplementary Material E.

### Statistical Analysis

To examine differences in loneliness and social closeness, independent samples *t*-tests were applied. In order to examine differences in the frequencies of virtual contacts and in-person contacts to non-household members, 2 × 2 rm-ANOVA with the between-subjects factor “group” (HC, BPD) and within-subject factor “channel” (virtual, in person), was conducted. Need for touch and importance of touch in relationships towards members of participants' social network were compared between groups with independent samples *t*-test. Ratings of pleasantness of the videos depicting different types of touch were analysed with a 2 × 2 × 2 rm-ANOVA with the between-subjects factor “group” (HC, BPD) and within-subject factors “social” (social, non-social) and “valence” (negative, positive).

The role of the appraisal of social touch as a moderator in the relationship of contact frequencies to feelings of social isolation, respectively to the experienced burden through social distancing, was investigated with three multiple regression analyses across the whole sample. Additionally to the pre-registered analysis of the relationship between contact frequencies and the experience of closeness to members of the participants' social network (hypothesis 2), we analysed this relationship between objective and subjectively experienced social isolation on a more global level based on network size and loneliness measured with the ULS-R. In the first model, we used the frequency of in-person contacts and virtual contacts, the three facets of the appraisal of social touch, as well as their interactions with the frequencies of in-person contacts and virtual contacts to predict the closeness experienced within the participant's social network. In the second model, we predicted the ULS-R score with SNI size, the three facets of the appraisal of social touch, that is the NFTPS score, the liking of positive social touch (estimated as the difference between liking of positive social and non-social touch), and the importance of touch within the SN, as well as their interactions with the SNI size. In the third model, we repeated the first analysis exploratively with the experienced burden through physical distancing as dependent variable. Since the “physical distancing” policies aimed to reduce in-person contacts with persons from other households, we excluded ratings related to individuals living within the same household when calculating the mean scores of contact frequencies and closeness in model one and three.

Associations of ACE, the capacity to feel close to others as one dimension of attachment, and appraisal of social touch in BPD were examined with multivariate multiple regression. Hereby, we calculated a regression model with the CTQ score together with attachment closeness and its interaction with CTQ as predictors for the three facets of the appraisal of social touch.

All analyses were performed using SPSS software (version 25) and R (version 4.0.4). The level of significance was set to α = 0.05. For regression analyses, predictor variables were z-transformed before analyses. Participants exhibiting scores ± 2.5 *SD* from the mean were excluded from the respective analysis as outliers and can be found in see Supplementary Table S1. The observed power remained sufficient. We adjusted *P*-values according to Benjamini and Hochberg (83) for *post-hoc* tests (sub-designs of the rm-ANOVA, as well as pairwise comparisons). None of our participants exhibited processing times faster than 60% of the median and all stated that they filled out the query honestly and alone. Please note that we used the median instead of the mean of processing time to adjust for longer breaks in single participants during answering the survey.

### Pre-registration

The main hypotheses were pre-registered together with the design and planned analyses (https://aspredicted.org/xs67c.pdf). In addition to our pre-registered hypotheses, we investigated the interplay of the contact frequencies, the appraisal of social touch and the experienced burden through social distancing. We did not fully achieve the sample size of 80 BPD and 80 HC participants planned in the pre-registration, for further details on the recruitment procedure, see Supplementary Material A.

## Results

### Loneliness and Closeness Within the Social Network

**Figure 1:**
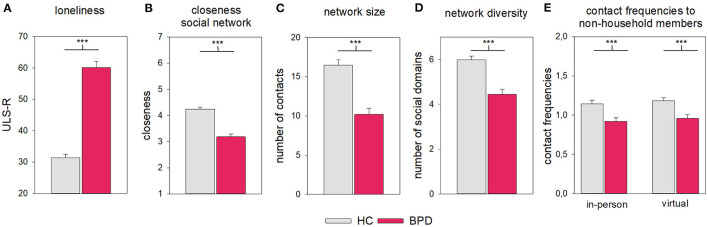
Social connectedness and features of the social network in the HC and BPD groups. **(A)** ULS-R, **(B)** closeness within the network, **(C)** network size and **(D)** diversity, and **(E)** contact frequencies. Error bars represent 1 SE. ***P < 0.001.

ULS-R scores revealed a higher level of loneliness in the BPD group compared to HCs [HC: *M* = 31.39, *SD* = 9.40, BPD: *M* = 60.17, *SD* = 14.29, *t* (99.52) = −13.30, *P* < 0.001, *d* = −2.41, Figure 1A]. When judging the closeness to members of their social network, individuals with BPD reported to feel less close than the HC group [HC: *M* = 4.24, *SD* = 0.66, BPD: *M* = 3.18, *SD* = 0.85, *t* (126) = 7.94, *P* < 0.001, *d* = 1.41, Figure 1B]. An exploratory analysis revealed that this was true for members of their social network with whom they do not live together, but not for members of the same household [non-household: HC: *M* = 3.96, *SD* = 0.73, BPD: *M* = 2.90, *SD* = 0.95, *t* (128) = 7.94, *P* < 0.001, *d* = 1.27; household: HC: *M* = 6.19, *SD* = 0.98,.BPD: *M* = 5.69, *SD* = 1.21, *t* (43.91) = 1.77, *P* = 0.083, *d* = 0.46]. For additional information on closeness in different social domains (Supplementary Table S2 and Supplementary Figure S1).

### Social Networks and Communication Channels

The social networks of the BPD group were smaller and less diverse than those of the HC group [size: HC: *M* = 16.45, *SD* = 5.68, BPD: *M* = 10.20, *SD* = 6.26, *t* (126) = 5.92, *P* < 0.001, *d* = 1.05; diversity: HC: *M* = 5.99, *SD* = 1.36, BPD: *M* = 4.45, *SD* = 1.72, *t* (127) = 5.66, *P* < 0.001, *d* = 1.00; Figures 1C,D].

When judging the frequency of contacts when using different communication channels, we restricted our analyses to members of the social network that do *not* live in the same household. The frequency of in-person contacts and virtual contacts did not differ significantly and this was true for both groups [Figure 1E, “channel:” *F*_(1, 126)_ = 1.62, *P* = 0.205, $\eta_{\text{p}}^{2}$ = 0.01; “group” × “channel”: *F*_(1, 126)_ < 0.01, *P* = 0.997, $\eta_{\text{p}}^{2}$ < 0.01; in person: HC: *M* = 1.14, *SD* = 0.38, BPD: *M* = 0.92, *SD* = 0.38, virtual: HC: *M* = 1.18, *SD* = 0.31, BPD: *M* = 0.96, *SD* = 0.34]. However, overall, contacts were less frequent in the BPD compared with the HC group [“group”: *F*_(1, 126)_ = 17.25, *P* < 0.001, $\eta_{\text{p}}^{2}$ = 0.12, HC: *M* = 1.16, *SD* = 0.29, BPD: *M* = 0.94, *SD* = 0.33]. For additional information for social domains, see Supplementary Tables S3, S4 and Supplementary Figures S2, S3.

### Appraisal of Social Touch

#### Need for Touch

**Figure 2:**
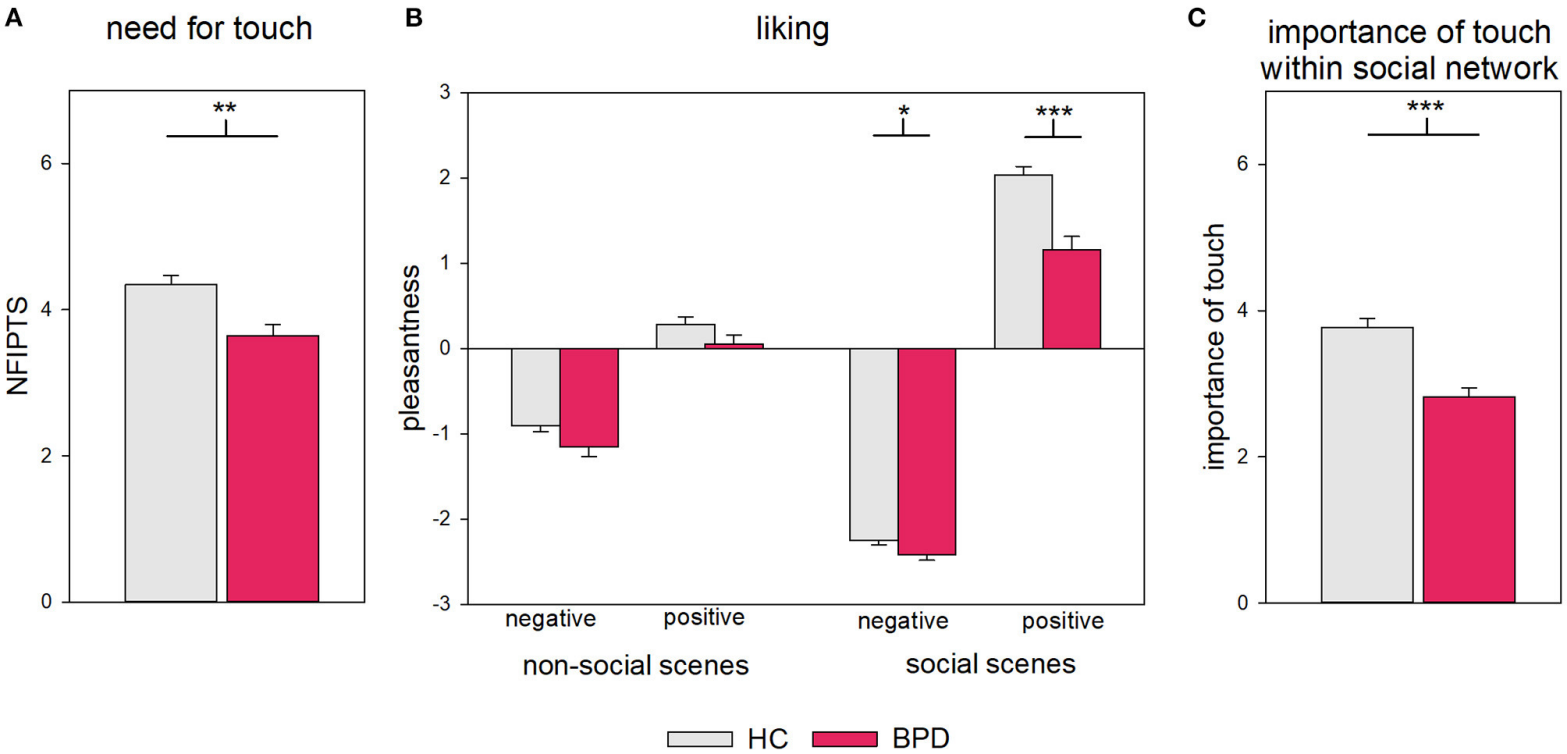
**(A)** Need for touch, **(B)** liking of touch, and **(C)** importance of touch within social network in HC and BPD. Error bars represent 1 SE. *P < 0.05. **P > 0.01. ***P < 0.001.

NFIPT scores indicate a lower need for social touch in the BPD group compared with the HC group [HC: *M* = 4.34, *SD* = 1.04, BPD: *M* = 3.64, *SD* = 1.26, *t* (116.88) = 3.49, *P* = 0.001, *d* = 0.61, Figure 2A].

#### Liking of Social Touch

Compared to HCs, BPD patients judged the video clips as less pleasant [“group:” *F*_(1, 118)_ = 100.05, *P* < 0.001, $\eta_{\text{p}}^{2}$ = 0.16]. The strength of differences between groups was influenced by both experimental factors [“valence” × “social” × “group:” *F*_(1, 118)_ = 12.09, *P* = 0.001, $\eta_{\text{p}}^{2}$ = 0.09, Figure 2B, for summary of ANOVA results see Supplementary Table S6]. A 2 × 2 ANOVA sub-design for social scenes revealed that the pleasantness ratings in the BPD group was stronger reduced for the positive than the negative social scenes compared with HC [“group” × “valence”: *F*_(1, 118)_ = 12.76, *P* = 0.001, *p*_*FDR*_ = 0.001, $\eta_{\text{p}}^{2}$ = 0.10; “valence”: *F*_(1, 118)_ = 1,544.96, *P* < 0.001, *p*_*FDR*_ < 0.001, $\eta_{\text{p}}^{2}$ = 0.93; “group”: *F*_(1, 118)_ = 28.25, *P* < 0.001, *p*_*FDR*_ < 0.001, $\eta_{\text{p}}^{2}$ = 0.19; comparison between groups: negative: HC: *M* = −2.25, *SD* = 0.44, BPD: *M* = −2.42, *SD* = 0.47, *t* (118) = 2.02, *P* = 0.046, *p*_*FDR*_ = 0.046 *d* = 0.28; positive: HC: *M* = 2.03, *SD* = 0.80, BPD: *M* = 1.15, *SD* = 1.17, *t* (87.63) = 4.68, *P* < 0.001, *p*_*FDR*_ < 0.001, *d* = 0.90]. In contrast, a 2 × 2 sub-design for non-social scenes revealed no statistically significant difference between groups in the effects of valence: all participants assessed negative scenes as less pleasant than positive scenes [“valence”: *F*_(1, 118)_ = 174.02, *P* < 0.001, *p*_*FDR*_ < 0.001, $\eta_{\text{p}}^{2}$ = 0.60; “group” × “valence”: *F*_(1, 118)_ =.01, *P* = 0.931, *p*_*FDR*_ = 0.931, $\eta_{\text{p}}^{2}$ < 0.0;1 “group”: *F*_(1, 118)_ = 6.66, *P* = 0.011, *p*_*FDR*_ = 0.011, $\eta_{\text{p}}^{2}$ = 0.19] For summary of ANOVA results of sub-designs, see Supplementary Tables S7, S8.

Please note that, in general, videos of positive touch were rated as more pleasant than videos of negative touch [“valence”: *F*_(1, 118)_ = 1029.19, *P* < 0.001, $\eta_{\text{p}}^{2}$ = 0.90]. This difference was higher for social compared to non-social scenes [“valence” × “social”: *F*_(1, 118)_ = 674.64, *P* < 0.001, $\eta_{\text{p}}^{2}$ = 0.85], with negative touch being rated as even more negative and positive touches as even more positive [pairwise comparison non-social with social: negative: *t* (119) = 20.64, *P* < 0.001, *p*_*FDR*_ < 0.001, *d*_*z*_ = 1.88; positive: *t* (119*)* = −16.64, *P* < 0.001, *p*_*FDR*_ < 0.001, *d*_*z*_ = −1.52].

#### Importance of Touch in Social Relationships

Individuals with BPD reported a lower importance of touch in their relationships [HC: *M* = 3.77, *SD* = 1.01, BPD: *M* = 2.82, *SD* = 0.96, *t* (128) = 5.46, *P* < 0.001, *d* = 0.96, Figure 2C]. An exploratory analysis revealed that this applied only for social network members outside of their household but not household members [household: HC: *M* = 6.10, *SD* = 0.93, BPD: *M* = 5.62, *SD* = 1.33, *t* (41.51) = 1.63, *P* = 0.111, *d* = 0.44; outside: HC: *M* = 3.43, *SD* = 1.10, BPD: *M* = 2.58, *SD* = 1.00, *t* (128) = 4.61, *P* < 0.001, *d* = 0.81]. For additional information on the importance of touch in different social domains, see Supplementary Table S9 and Supplementary Figure S4.

### Interpersonal Touch as a Moderator Between the Frequency of Social Contacts and Social Closeness Towards Members of the Social Network Living Outside of the Own Household

**Figure 3:**
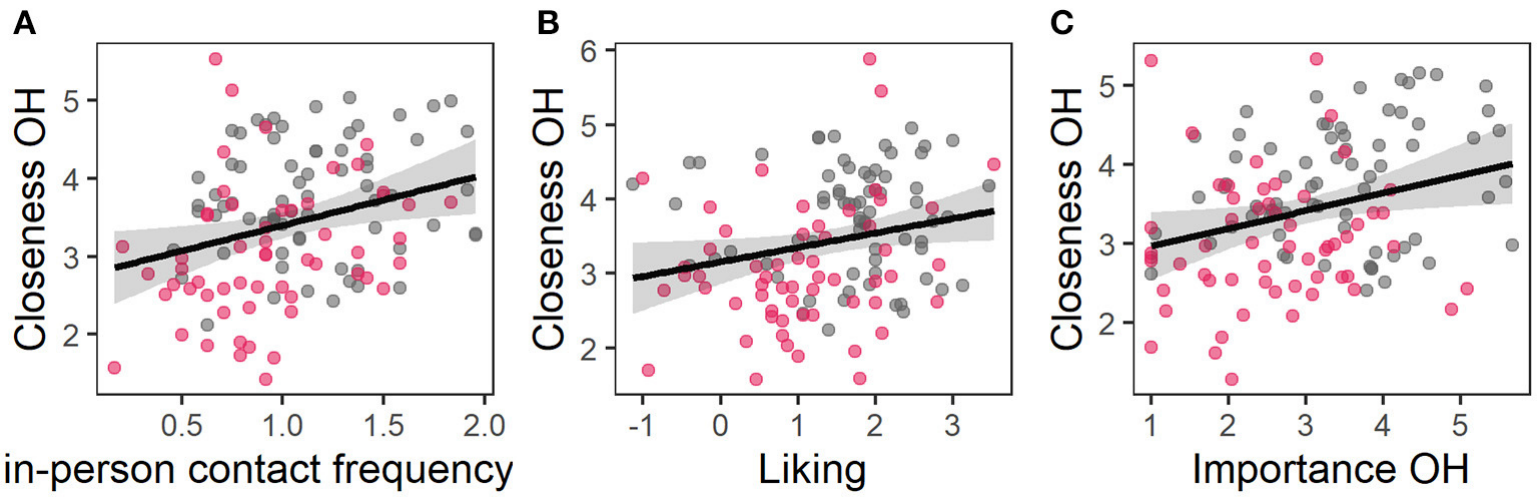
Partial residual plots of regression model 1; the observed data is based on the values of **(A)** in-person contact frequency, **(B)** liking, and **(C)** importance of touch and the model error. The grey area represents the confidence interval, grey dots data of HC and red dots data of BPD.

Multiple regression analyses revealed that contact frequencies and appraisal of social touch predicted 29.8% of the variance of closeness to members of the social network outside of the own household [*F*_(11, 114)_ = 4.39, *P* < 0.001, adjusted *R*^2^ = 0.23, Table 2]. With increasing frequency of in-person contacts to non-household members, as well as a higher liking and importance of social touch in relationships to non-household members, the experienced closeness towards these members of the social network increased (Figure 3). None of the interactions of the frequency of in-person contacts and virtual contacts with the facets of the appraisal of social touch were significant predictors.

**Table 2:** Prediction of social closeness by contact frequencies and appraisal of social touch.

	**β**	***SE***	***t***	***P*-Value**	
Intercept	3.43	0.08	41.35	<0.001	***
In-person-OH	0.26	0.10	2.62	0.010	**
Virtual-OH	0.14	0.10	1.38	0.169	
Need for touch	−0.11	0.12	−0.90	0.369	
Liking of touch	0.19	0.09	2.21	0.029	*
Importance of touch-OH	0.25	0.11	2.36	0.020	*
In-person-OH * need	0.03	0.14	0.24	0.809	
In-person-OH * liking	−0.08	0.12	−0.67	0.506	
In-person-OH * importance-OH	−0.07	0.13	−0.52	0.604	
Virtual-OH * need	0.28	0.17	1.68	0.096	
Virtual-OH * like	−0.04	0.11	−0.39	0.697	
Virtual-OH * importance-OH	−0.08	0.14	−0.54	0.588	

OH, members outside the own household. *p < 0.05, **p < 0.01, **p < 0.001.

### Interpersonal Touch as a Moderator Between Social Network Size and Loneliness

**Figure 4:**
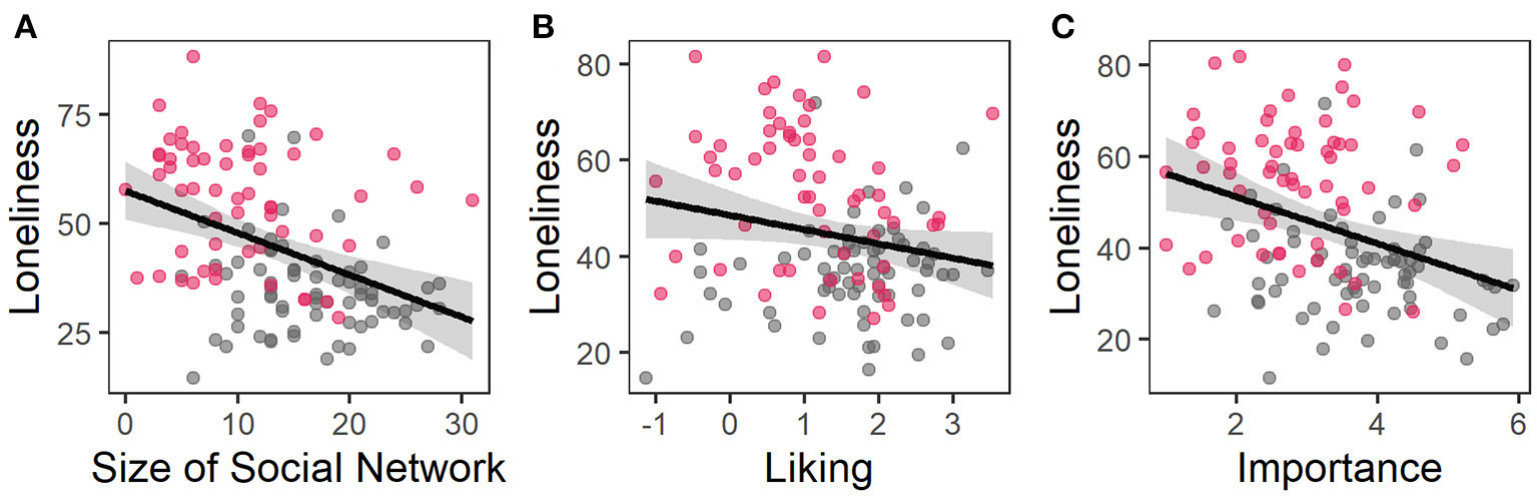
Partial residual plots of regression model 2; the observed data is based on the values of **(A)** size of social network, **(B)** liking, and **(C)** importance of touch and the model error. The grey area represents the confidence interval, grey dots data of HC and red dots data of BPD.

Multiple regression analyses revealed that network size and appraisal of social touch predicted 35.4% of the variance of loneliness [*F*_(7, 117)_ = 9.16, *P* < 0.001, adjusted *R*^2^ = 0.32, Table 3]. With an increase of the network size, as well as a stronger liking and importance of touch the ULS-R score decreased (Figure 4). None of the interactions of the social network size with the facets of the appraisal of social touch were significant predictors.

**Table 3:** Prediction of loneliness (ULS-R) by social network size and appraisal of social touch.

	**β**	***SE***	***t***	***P*-Value**	
Intercept	44.53	1.49	29.90	<0.001	***
SNI size	−6.49	1.58	−4.10	<0.001	***
Need for touch	0.27	1.96	0.14	0.889	
Liking of touch	−2.96	1.49	−1.99	0.049	*
Importance of touch	−5.63	1.76	−3.20	0.002	**
SNI size * need for touch	−0.84	2.12	−0.40	0.692	
SNI size * liking of touch	−0.94	1.58	−0.60	0.552	
SNI size * importance of touch	1.65	1.82	0.91	0.366	

Predictors were z-standardised. *p < 0.05, **p < 0.01, **p < 0.001.

### Interpersonal Touch as a Moderator Between the Frequency of Social Contacts Towards Members of the Social Network Living Outside of the Own Household and Burden Through “Physical Distancing”

**Figure 5:**
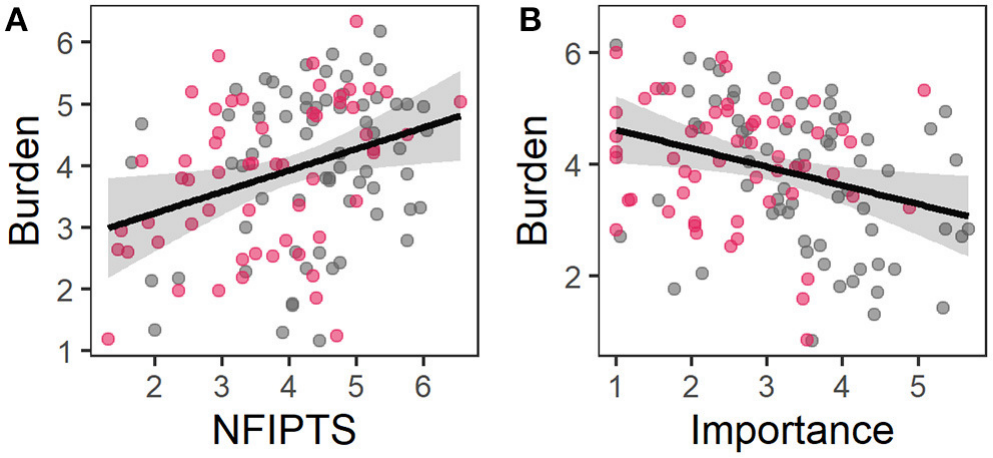
Partial residual plots of regression model 3; the observed data is based on the values of **(A)** need for interpersonal touch scale and **(B)** importance of touch and the model error. The grey area represents the confidence interval, grey dots data of HC and red dots data of BPD.

Multiple regression analysis revealed that contact frequencies and appraisal of social touch explained 19.0% of the variance of experienced burden through “physical distancing” [*F*_(11, 113)_ = 2.40, *P* = 0.010, adjusted *R*^2^ = 0.11, Table 4]. Contact frequencies did not predict the experienced burden through “physical distancing” policies, but a higher need for touch and a lower importance of touch predicted a higher burden (Figure 5).

**Table 4:** Prediction of burden through physical distancing policies by contact frequencies and appraisal of social touch.

	**β**	***SE***	***t***	***P*-Value**	
Intercept	3.94	0.12	33.74	<0.001	***
In-person-OH	0.17	0.14	1.26	0.209	
Virtual-OH	0.13	0.14	0.90	0.368	
Need for touch	0.41	0.16	2.46	0.015	*
Liking of touch	0.03	0.12	0.24	0.807	
Importance of touch-OH	−0.38	0.15	−2.49	0.014	*
In-person-OH * need	−0.25	0.20	−1.23	0.220	
In-person-OH * liking	0.18	0.17	1.05	0.296	
In-person-OH * importance-OH	0.10	0.18	0.58	0.562	
Virtual-OH * need	−0.29	0.23	−1.22	0.225	
Virtual-OH * like	−0.03	0.15	−0.20	0.840	
Virtual-OH * importance-OH	0.16	0.20	0.82	0.413	

OH, members outside the own household. *p < 0.05, **p < 0.001.

Please note that both groups did neither differ in the burden induced by social distancing measures nor in their compliance with these behavioural recommendations [burden: HC: *M* = 3.93, *SD* = 1.27, BPD: *M* = 3.76, *SD* = 1.30, *t* (127) = 0.72, *P* = 0.474; compliance HC: *M* = 4.73, *SD* = 0.97, BPD: *M* = 4.87, *SD* = 0.85, *t* (123) = −0.84, *P* = 0.404].

### ACE, Attachment Closeness and the Appraisal of Interpersonal Touch in BPD

To investigate whether the severity of ACE influences the appraisal of social touch in the BPD group, we calculated a multivariate linear regression model with the CTQ score and attachment closeness as well as its interaction with CTQ score as predictors and the need, liking and importance of social touch as dependent variables. The analyses revealed that the CTQ score predicted neither the need, liking nor the importance of social touch (all *P* > 0.1). A higher attachment closeness predicted a higher need for touch and a higher liking of touch, but not a higher importance of interpersonal touch towards the members of the social network. It did not moderate the relation between the severity of ACE and the appraisal of social touch. For further details, see Supplementary Tables S10, S11.

## Discussion

In the present study, our findings revealed differences between individuals with BPD and HCs regarding their subjective feelings and their objective level of social isolation as well as their appraisal of social touch. Participants of the BPD group reported to feel more lonely and less close to those members of their social networks living without the same household. Moreover, their social networks were smaller and less diverse with a lower frequency of both in-person contacts and virtual contacts with members of their social network living outside their household. Social touch as one fundamental component of social relationships had an altered relevance in BPD in all three investigated facets: individuals with BPD reported a lower need for touch, a lower liking of particularly positive interpersonal touch and a lower importance of touch in the relationships at least towards those members of their social networks living outside their own household. Investigating the interplay between subjective and objective isolation and appraisal of social touch revealed that being objectively more isolated to others as well as a lower liking and lower importance of social touch during social interactions predicted feeling socially isolated. This was true for both, analysing these associations on a general level as well as when investigating this interplay for the participants' actual social networks. Similarly, the burden experienced during “physical distancing” policies was predicted by the evaluation of the role of social touch. However, in contrast to feeling lonely, both groups experienced a comparable burden and beyond the importance of social touch, it was particularly the need for touch instead of the liking of touch that predicted the severity of the burden people experience during the COVID-19 pandemic. When analysing the underlying mechanism, we did not find a history of ACE to predict the appraisal of social touch. In contrast, a higher capacity to feel close to others as one dimension of attachment was associated with a higher need for touch and stronger liking of positive social touch.

### Objective and Subjectively Experienced Social Isolation

Compared to HCs, individuals with BPD reported both, feeling lonelier overall, but also feeling less close to members of their social network. This is in line with our hypotheses and confirms previous findings of increased levels of loneliness in BPD in general (5–7) as well as reduced feelings of being socially included in experimental paradigms (84–86). Also, in line with our expectations and previous literature, compared to HCs, individuals with BPD reported smaller and less diverse social networks (6, 7, 10, 11). Our findings also emphasise the particular importance of meeting others in person. Only the frequency of in-person contacts, but not virtual contacts were shown to contribute to the feeling of closeness.

### Appraisal of Social Touch

Individuals with a history of BPD differed from HCs in all three assessed facets of their appraisal of social touch. As predicted and in line with literature (62), our data revealed not only a lower liking of pleasant touch in the BPD group, but also identified positive social touch as the domain especially affected in BPD. This is in line with prior findings about a negative bias in decoding positive social cues in BPD (87, 88). Moreover, the BPD group stated that social touch is less important in their relationships towards members of their social network, which fits with previous findings that they prefer greater interpersonal distance (41). Beyond facets of physical proximity investigated in previous studies in BPD, our findings suggest that there is also a lower need for social touch in BPD. One might speculate whether a lower need and a lower importance of social touch are consequences of a lower liking of positively annotated social touch. However, the lower need for touch might alternatively be explained by an individual's denial of this need due to fears associated with physical proximity or shame (25). In this case, another speculative explanation could be that impulsive sexual behaviour seen in some patients with BPD constitutes a maladaptive attempt to satisfy an unmet need for social touch (89, 90). Liking and living affectionate touch to a lower extent than healthy individuals, might also constitute a source for misunderstandings in social relationships, particularly with people who value physical closeness and use touch to provide social support and form interpersonal bonds. Our findings emphasise the need for further studies in BPD investigating the interplay between these different facets of social touch, as well as their effects on impairments of forming, maintaining and benefitting from social relationships.

#### Appraisal of Social Touch and Social Isolation

A higher liking of positive touch and a higher importance of social touch during social interactions predicted lower levels of social isolation, supporting the role of touch for closeness. However, in contrast to our hypotheses, neither the need, liking nor the importance of touch moderated the relationship between objective and subjectively experienced social isolation. Together with the finding that the frequency of virtual contact was not a predictor of feeling socially isolated, this implies that in-person encounters are beneficial regardless of the possibility to satisfy the need for social touch. Possible reasons could be that meeting others in person allows in a larger extent for joined activities and might mostly, for example a walk, take longer and thus allow a deeper and potentially emotionally more supportive exchange. In addition to being able to see a person's head-to-toe body language and posture when meeting personally, touch could also be helpful as a further social cue to stabilise and deepen social relationships and thus be beneficial for the feeling of social closeness.

#### Appraisal of Social Touch and Burden Through “Physical Distancing”

In addition to the preregistered main analyses, we investigated whether contact frequencies and the appraisal of social touch are associated with the experience of social distancing policies during the COVID-19 pandemic. Contrary to the suggestion of Preti, Di Pierro (3), individuals with BPD did not differ from HCs in terms of burden through and compliance with social distancing policies. The impairments in interpersonal and emotional domains in BPD did not seem to affect them additionally in dealing with the challenges and consequences of social distancing. However, a high percentage of BPD patients in this study were currently receiving therapeutic treatment. This might be one possible reason for the finding that no extraordinary burden was induced by the “physical distancing” rules, if these individuals had already learnt strategies for coping with loneliness and also received therapeutic support during the pandemic. Another reason could be that due to BPD patients' smaller social networks they experience a smaller change in their social life. Contrary to our hypothesis, the burden through social distancing was not predicted by contact frequencies and this was not modulated by the appraisal of social touch. However, in line with our hypotheses, a higher need for touch constitutes a vulnerability factor during “physical distancing”. Higher need for touch was associated with higher burden, although independently from objective social isolation. In contrast, a higher burden was also predicted by a lower instead of a higher importance of touch. This finding suggests—together with the association of a higher importance of touch with higher levels of experienced closeness—that the existence of close relationships supported by social touch have a protective effect and are less challenged by a transient state during which in-person meetings are prohibited. Whether this holds true when “physical distancing” recommendations are in place for extended periods of time, has to be investigated during the further course of the COVID-19 pandemic.

#### Appraisal of Social Touch, ACE, and Attachment Style

Contrary to our hypotheses, the severity of childhood trauma did not predict the appraisal of social touch in the BPD group, that is, neither the need, liking nor importance of social touch. However, it can be assumed that the impact of childhood trauma on the appraisal of social touch depends on whether the trauma was related to a lack of physical contact or aversive experiences with social touch (47, 48). In the present study, individuals with BPD reported primarily emotional neglect and abuse as types of trauma. While our findings do not support the role of trauma in alterations of the appraisal of social touch in BPD, further studies are needed to investigate whether there might be associations found in those who suffered especially from trauma associated with social touch such as physical or sexual abuse or physical neglect. Independently from the severity of childhood trauma, attachment closeness predicted a higher need and liking of positive touch, but not a higher importance of touch in actual interpersonal relationships. These findings suggest that the need for and liking of touch correspond to the bodily expressions of the capacity to feel close to others. In contrast, the importance of touch in relationships with members of the social network might be influenced more strongly by other factors such as the social domain and the related importance of feeling close.

### Limitations of the Current Study

The present study has some limitations. Some refer to the investigated sample. Due to the overrepresentation of female BPD patients in the health system, we included female participants only. Results can therefore not be generalised to men; we therefore emphasise the importance of replicating this study in a more gender-diverse sample. Moreover, individuals of the BPD group had been formally diagnosed with BPD in the past. BPD participants were included in the study based on the current severity of BPD features rated on PAI-BOR without reassessing their former BPD diagnosis. Due to this group differences might be larger than reported, since some of the BPD participants might not fulfil clinical criteria anymore. However, sample diversity might also resemble diverse BPD presentation. BPD is characterised by frequent alterations between recovery and the reoccurrence of symptoms, which is often linked to a persistence of low levels of social functioning and impairments in social cognition during remission and recovery (87, 91–93). This shows the importance of identifying factors that might contribute to the fluctuation of severity in the course of the disorder. Finally, we did not include a healthy control group with exposure to childhood trauma. Therefore a differentiation of the effects of childhood trauma and borderline personality psychopathology was not possible.

Due to the COVID-19 pandemic, we collected data through online surveys. Hence, we focussed on self-report questionnaires. Please note that it cannot be ruled out that these self-report measures might be influenced differentially in both groups by a bias due to socially desirable answers. Further studies are needed extending the measures we used by experimental paradigms using actual touch in addition to rating video clips. While video clips may still be better at simulating the sensual aspect of touch compared to verbal descriptions of scenarios, it falls short of using actual (social) touch. Moreover, further studies are needed using experimental tasks combined with psychophysiological and neural correlates of processing to investigate the different functions of social touch (e.g., communicating social support in distressing situations or showing affection towards others) within different social domains (romantic partners, family, close friends or work colleague). In addition, analysis approaches like hierarchical linear models might be useful for future studies in order to investigate the findings of our ANOVA on the individual level in more detail.

When analysing the relationship of contact frequencies and the appraisal of touch to subjective social isolation and burden trough social distancing, we calculated the regression analyses across the entire sample to cover a broad range of evaluations of loneliness, the appraisal of touch and social isolation. Since both groups differ in these variables, our results might be confounded by group membership although the partial correlation plots suggest that the association are not only driven by differences between groups. Nevertheless, further studies with larger sample sizes are needed that allow to include a diagnosis with BPD as an additional factor in the analyses and take interactions between this factor and the other predictors into account. Moreover, future studies may investigate, whether these associations can be found across larger groups of healthy individuals covering the broad spectrum of loneliness and appraisal of social touch to investigate the interplay between these factors independently of BPD.

### Conclusions

Our study contributes to the understanding of impairments of social belonging in BPD by identifying the appraisal of social touch beyond social isolation as a factor associated with the experience of loneliness and reduced closeness towards others. Our findings suggest that it is important to consider different facets of the appraisal of social touch when researching their effect on social isolation as well as perceived burden due to “physical distancing” measures.

## Data Availability Statement

The datasets presented in this article are not readily available because according to European law (GDPR), data containing potentially identifying or sensitive patient information are restricted; our data involving clinical participants are not freely available in the article, Supplementary Material, or in a public repository. Data access can be requested on reasonable demand *via* the corresponding author. Requests to access the datasets should be directed to anna.schulze@zi-mannheim.de.

## Ethics Statement

The studies involving human participants were reviewed and approved by Research Ethics Board II of the Medical Faculty Mannheim of Heidelberg University. The patients/participants provided their written informed consent to participate in this study.

## Author Contributions

AS, MBi, KA, and SL designed the study. AS and LW set up the experiment. FU, LW, and AS recruited the sample. AS conducted all statistical analyses and created the figures. SL, MBi, and MBo provided substantive and conceptual feedback on all draft. All authors contributed to and have approved the final manuscript.

## Funding

This study was funded by the Deutsche Forschungsgemeinschaft (DFG, German Research Foundation)—GRK2350/1–324164820.

## Conflict of Interest

The authors declare that the research was conducted in the absence of any commercial or financial relationships that could be construed as a potential conflict of interest.

## Publisher's Note

All claims expressed in this article are solely those of the authors and do not necessarily represent those of their affiliated organizations, or those of the publisher, the editors and the reviewers. Any product that may be evaluated in this article, or claim that may be made by its manufacturer, is not guaranteed or endorsed by the publisher.
