# Supplementary material for: Social Touch, Social Isolation, and Loneliness in Borderline Personality Disorder During the COVID-19 Pandemic
Source: Front Psychiatry. 2022 Jun 23;13:876413. doi: 10.3389/fpsyt.2022.876413 (PMC9260178; doi:10.3389/fpsyt.2022.876413)
Supplement: Supplementary file 1 [file Data_Sheet_1.PDF]

Supplementary Material

A. Description of Recruitment Procedure

From the KFO data base, we contacted 145 healthy women (HC) and 132 women who had met a DSM-IV diagnosis of Borderline Personality Disorder (BPD) within the last six years. Fifty-three individuals (32 HC, 21 BPD) did not respond and 75 individuals (43 HC, 32 BPD) were not interested in participation or did not provide their written informed consent. Of the 149 participants (70 HC, 79 BPD), we excluded 18 individuals of the BPD group from analyses since 3 individuals did not fill in all questionnaires and 15 reported a severity of BPD features below the cut-off score for a clinically relevant level of BPD features (VEI-BOR<38). In addition, one HC participant was excluded, because she did not fill in all questionnaires. Thus, the final dataset consisted of 130 participants (69 HC, 61 BPD). Please note that we contacted only HCs without a history of childhood traumatization (CTQ score below cut-offs for a female community sample; 1, please see below).

B. Description of the Self-Report Questionnaires for Psychopathology and Childhood Traumatization

Severity of Borderline Symptoms was assessed with the short version of the Borderline Symptom List (BSL-23; 2). Participants were asked to indicate how strong they experienced 23 symptoms within the last week on a 5-point Likert-scale from 0 (*not at all*) to 4 (*very strong*). The mean score ranges from 0 to 4 with higher scores indicating a higher symptom severity. In the present study internal consistency for the BSL-23 was α = .97 (BPD: Cronbach's α = .93; HCs: Cronbach's α = .87).

We measured features of personality pathology that are associated with the borderline personality disorder by the Borderline Scale from the Personality Assessment Inventory (PAI-BOR: 3, German version: 4). The scale consists of 24 items answered on a 4-point Likert scale (0 – 3; total range from 0 - 72 with higher scores indicating higher BPD features). Internal consistency for the VEI-BOR was α = .95 (BPD: Cronbach's α = .85; HC: Cronbach's α = .79).

Severity of depressive symptoms was measured with Beck's Depression Inventory II (BDI-II; 5, German version: 6; range: 0-63; Cronbach's α in the current study were α = .96 for the total sample, α = .94 in the BPD and α = .87 in HC sample).

Severity of childhood trauma was assessed using the short form of the Childhood Trauma Questionnaire (CTQ-SF; 7, German version: 8). Subjects rate the frequency of maltreatment in childhood and adolescence on five subscales with five items each, using a 5-point Likert scale. Items are combined to form five subscales ranging from 5 to 25 that assess the frequency of emotional abuse (BPD: Cronbach's α = .85), emotional neglect (BPD: Cronbach's α = .93), physical abuse (BPD: Cronbach's α = .87), physical neglect (BPD: Cronbach's α = .69), and sexual abuse (BPD: Cronbach's α = .98). Total scores range from 25 to 125. Cronbach's alpha for the total score in the BPD group of the current study was α = .94. Please note that CTQ data was obtained from previous study participation in the KFO-256.

C. List of Questions about the Use of Different Communication Channels

How have you been in contact with … in the last four weeks?
in the same household
personal meetings
via telephone
in writing via electronic media (e.g. messenger, mail)
via video telephony (e.g. Zoom, Skype)
via social media (e.g. facebook, twitter, Instagram)
via letters

D. List of Questions about the Relevance of Interpersonal Touch towards Members of the Social Network

How important is interpersonal touch to you in your relationship with ...?
... when we greet each other or say goodbye.
... when I want to comfort, reassure or support the other person.
... when the other person wants to comfort, reassure or support me.
... when I want to show the other person that I pay attention to him/her.
... when the other person shows me that he or she wants to pay attention to me.
... just like that, for no apparent reason.

E. List of the Social Distancing-Related Questions

Ratings for the questions related to ‘social distancing’ were averaged (overall Cronbach’s α =.89; BDP: Cronbach’s α = .88; HC: Cronbach’s α = .90).

How burdening is it for you to comply to the following rules:
Keep 1.5m distance
Avoid public places
Carry out only necessary routes
Renounce private travelling
Stay as much at home as possible
Avoid meetings with persons from more than two households
Reduce private social contacts

Table S1

Number of cases excluded as outliers in our analyses

Analysis	HC	BPD
3.1 t-test ULS-R	0	1
3.1 t-test closeness	0	2
3.1 t-test closeness household	0	1
3.1 t-test closeness non-household	0	0
3.2 t-test network size	2	0
3.2 t-test network diversity	0	1
3.2 ANOVA contact frequencies	0	2
3.3.1 t-test need for touch	0	0
3.3.2 ANOVA liking of social touch	2	8
3.3.3 t-test importance of touch	0	0
3.3.3 t-test importance of touch household	0	2
3.3.3 t-test importance of touch non-household	0	0
3.4 multiple regression ULS-R	2	3
3.5 multiple regression closeness non-household	0	4
3.6 multiple regression burden	1	4
3.7 hierarchical multivariate multiple regression	-	4

Table S2

Social Closeness in Different Social Domains

domain	HC		BPD				
	M (SD)	*n*	*M (SD)*	*n*		*p*	*r*
partner	5.98 (1.20)	50	4.77 (1.98)	39	*Z* = -3.02	.003	.320
children	6.63 (0.76)	19	5.10 (0.88)	10	*U* = 16.50	<.001	.720
parents	5.50 (1.42)	68	3.15 (1.76)	59	*Z* = -5.81	<.001	.515
parents in law	3.64 (1.48)	42	2.71 (1.46)	28	*Z* = -2.49	.013	.298
relatives	4.60 (1.38)	62	3.12 (1.53)	42	*Z* = -4.53	<.001	.444
friends	4.42 (1.51)	69	3.53 (1.62)	55	*Z* = -2.89	.004	.259
parishioners	2.57 (1.45)	14	4.00 (0.00)	1			
classmates	3.20 (1.08)	15	2.64 (1.71)	22	*Z* = -1.39	.164	.229
work colleagues	3.42 (1.31)	62	3.06 (1.51)	36	*Z* = -1.12	.265	.113
neighbors	2.70 (1.59)	69	2.13 (1.52)	61	*Z* = -2.22	.026	.195
volunteering	3.50 (1.52)	6	3.14 (1.41)	14	*U* = 37.50	.718	.085
other groups	2.90 (1.17)	20	1.85 (1.21)	13	*Z* = -2.41	.016	.419
household	6.19 (0.98)	45	5.24 (1.78)	29	*Z* = -2.31	.021	.269
non-household	3.96 (0.73)	69	2.90 (0.95)	61	*Z* = -6.24	<.001	.547

Note. Comparison include different sample sizes and statistical analyses have to be interpreted with care due to the restricted sample size for some comparisons.

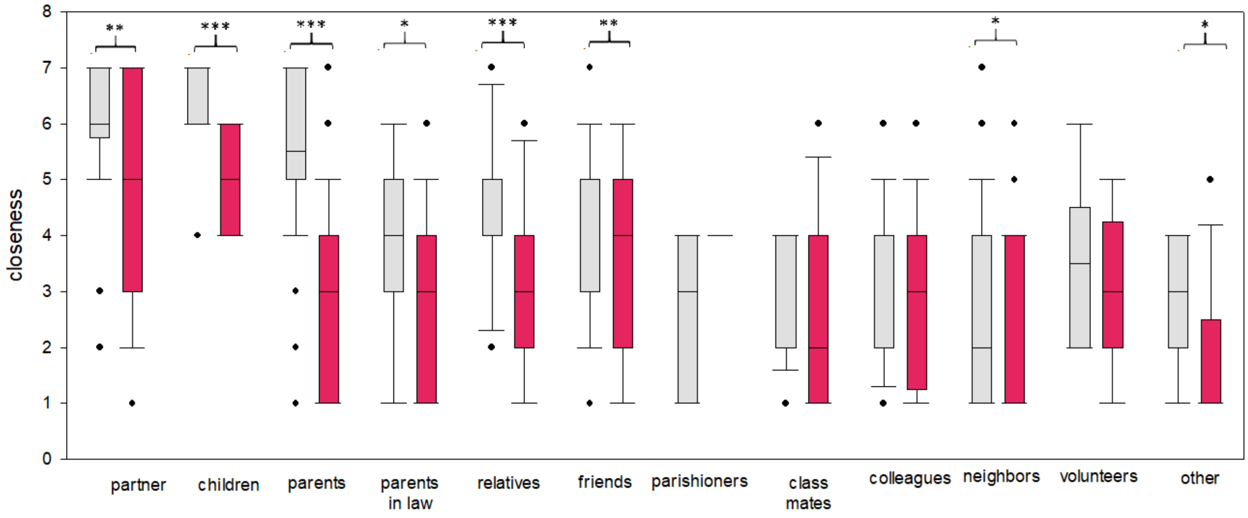

Figure S1. Social Closeness in different Social Domains.

Note: Comparison include different sample sizes and statistical analyses have to be interpreted with care due to the restricted sample size for some comparisons.

Table S3

Frequencies of contacts in person in Different Social Domains

domain	HC			BPD					
	M	*(SD)*	*n*	*M*	*(SD)*	*n*		*p*	*r*
partner	6.25	1.28	50	5.69	1.51	39	Z = -2.31	.021	.245
children	6.32	1.60	19	5.70	2.11	10	*U* = 77.00	.429	.195
parents	3.07	1.80	68	2.09	1.35	59	*Z* = -3.35	.001	.297
parents in law	1.82	1.36	42	1.63	0.78	28	*Z* = -0.50	.621	.060
relatives	2.23	1.32	62	1.77	1.06	42	*Z* = -1.75	.080	.172
friends	2.16	1.15	69	2.07	1.05	55	*Z* = -0.42	.673	.038
parishioners	1.04	0.04	14	1.00		1			
classmates	1.30	0.53	15	1.36	0.74	22	*U* = 164.00	.510	.007
work colleagues	2.54	1.81	62	2.43	1.52	36	*Z* = -0.12	.903	.012
neighbors	1.91	1.35	69	1.62	0.99	61	*Z* = -1.08	.282	.095
volunteering	1.00	0.00	6	1.64	0.93	14	*U* = 21.00	.091	.455
other groups	2.18	0.80	20	1.75	0.85	13	*Z* = -0.27	.790	.047
household	6.60	0.79	45	6.59	0.68	29	*Z* = -0.45	.656	.052
non-household	1.14	0.38	69	0.92	0.37	61	*Z* = -3.16	.002	.277

Note. Comparisons include different sample sizes and statistical analyses have to be interpreted with care due to the restricted sample size for some comparisons.

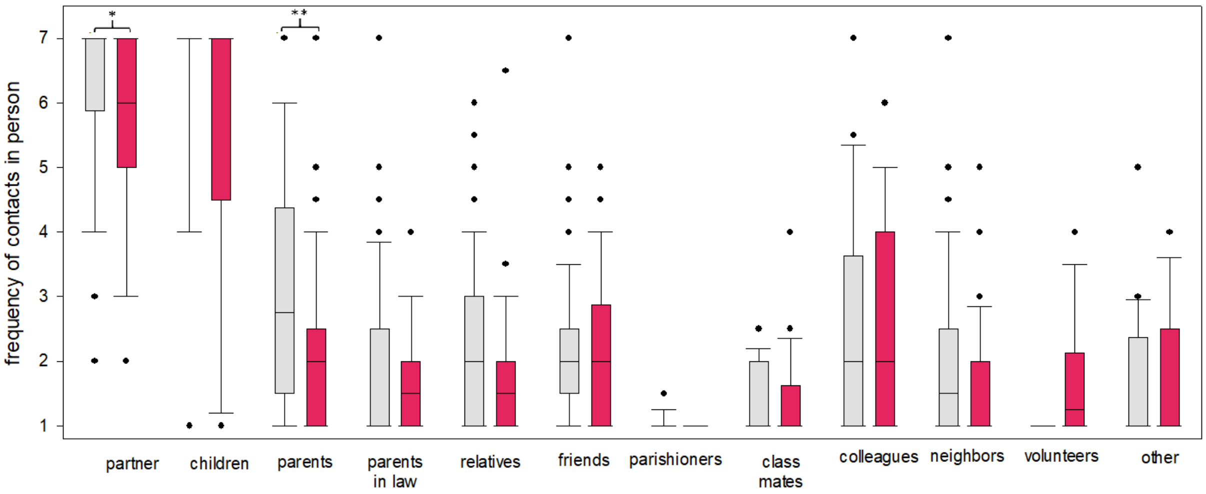

Figure S2. Frequencies of contacts in person in different Social Domains.
Note. Comparisons include different sample sizes and statistical analyses have to be interpreted with care due to the restricted sample size for some comparisons.

Table S4

Virtual Contact Frequencies in Different Social Domains

domain	HC			BPD					
	M	*(SD)*	*n*	*M*	*(SD)*	*n*		*p*	*r*
partner	2.92	1.28	50	5.69	1.01	39	Z = -0.03	.974	.003
children	1.90	1.02	19	2.18	1.15	10	*U* = 79.00	.484	.141
parents	2.55	0.73	68	2.04	0.77	59	*Z* = -3.84	< .001	.341
parents in law	1.51	0.55	42	1.60	1.04	28	*Z* = -0.09	.931	.010
relatives	2.35	0.89	62	2.27	1.00	42	*Z* = -0.69	.493	.067
friends	2.84	0.93	69	2.68	0.93	55	*Z* = -1.07	.285	.096
parishioners	1.13	0.43	14	1.00		1			
classmates	1.93	0.66	15	1.97	0.79	22	*U* = 165.00	.503	.000
work colleagues	2.97	1.27	62	2.49	1.11	36	*Z* = -1.70	.090	.171
neighbors	1.40	1.05	69	1.36	1.07	61	*Z* = -1.41	.414	.124
volunteering	1.70	0.64	6	2.24	1.14	14	*U* = 29.00	.312	.241
other groups	2.18	0.80	20	1.75	0.85	13	*Z* = -1.69	.092	.293
household	2.52	0.81	45	2.43	0.88	29	*Z* = -0.45	.653	.052
non-household	1.18	0.31	69	1.00	0.42	61	*Z* = -3.23	.001	.283

Note. Comparisons include different sample sizes and statistical analyses have to be interpreted with care due to the restricted sample size for some comparisons.

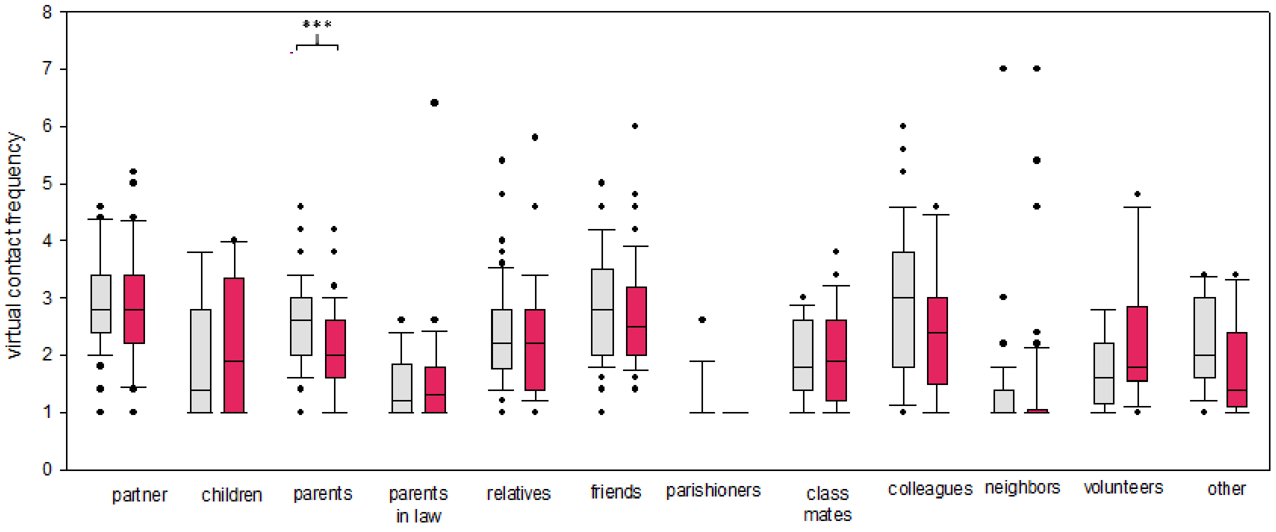

Figure S3. Virtual Contact Frequencies in different Social Domains.

Note. Comparisons include different sample sizes and statistical analyses have to be interpreted with care due to the restricted sample size for some comparisons.

Table S5

Contact frequencies in different communication cannels

channel	HC	BPD			
	M (SD)	*M (SD)*	*t*	*p*	*d*
personal meeting	2.75 (.70)	2.36 (.63)	3.40	.001	.597
phone call	3.02 (.71)	2.48 (.68)	4.45	< .001	.782
message	3.76 (.95)	3.53 (.83)	1.47	.144	.259
video call	1.99 (.75)	1.71 (.73)	2.16	.033	.380
social media	1.77 (.85)	1.63 (.79)	0.95	.346	.166
letter	1.09 (.30)	1.16 (.52)	-0.95	.343	-.168

Table S6

Results of the 2×2×2-ANOVA for Pleasantness Ratings of Touch Video Scenes

	F	*p*		η_p^2
gr	100.05	< .001	***	.16
social	1.33	.251		.01
social * gr	7.34	.008	**	.06
valence	1029.19	< .001	***	.90
valence * gr	4.76	.031	*	.04
social * valence	674.64	< .001	***	.85
social * valence * gr	12.09	.001	**	.09

Table S7

Results of 2x2-ANOVA Sub-Design Social Scenes

	F	*p*		η_p^2
gr	28.25	< .001	***	.19
valence	1544.96	< .001	***	.93
valence * gr	12.76	.001	**	.10

Table S8

Results of 2x2-ANOVA Sub-Design Non-Social Scenes

	F	*p*		η_p^2
gr	6.66	.011	*	.05
valence	174.02	< .001	***	.60
valence * gr	0.01	.931		< .01

Table S9

Importance of touch in different social domains

domain	HC		BPD				
	M (SD)	*n*	*M (SD)*	*n*		*p*	*r*
partner	6.20 (0.98)	50	6.03 (1.22)	39	*Z* = -0.61	.545	.064
children	6.33 (0.83)	19	4.60 (1.58)	10	*U* = 37.00	.007	.508
parents	4.73 (1.61)	68	2.61 (1.59)	59	*Z* = -6.26	< .001	.556
parents in law	3.02 (1.92)	42	1.89 (1.21)	28	*Z* = -2.42	.016	.289
relatives	4.70 (1.51)	62	3.20 (1.85)	42	*Z* = -4.16	< .001	.408
friends	4.80 (1.59)	69	4.26 (1.81)	55	*Z* = -1.64	.100	.148
parishioners	2.10 (1.22)	14	1.00 (0.00)	1			
classmates	2.62 (1.41)	15	1.99 (1.23)	22	*Z* = -1.41	.159	.231
work colleagues	2.01 (1.23)	62	1.61 (1.00)	36	*Z* = -1.94	.052	.196
neighbors	1.47 (1.07)	69	1.22 (0.66)	61	*Z* = -1.33	.183	.117
volunteering	1.86 (0.87)	6	1.99 (1.42)	14	*U* = 38.00	.779	.079
other groups	2.89 (1.42)	20	1.56 (0.91)	13	*Z* = -2.31	.021	.401
household	6.10 (0.93)	45	5.34 (1.67)	29	*Z* = -1.84	.067	.213
non-household	3.44 (1.10)	69	2.58 (1.00)	61	*Z* = -4.28	< .001	.497

Note. Comparisons include different sample sizes and statistical analyses have to be interpreted with care due to the restricted sample size for some comparisons.

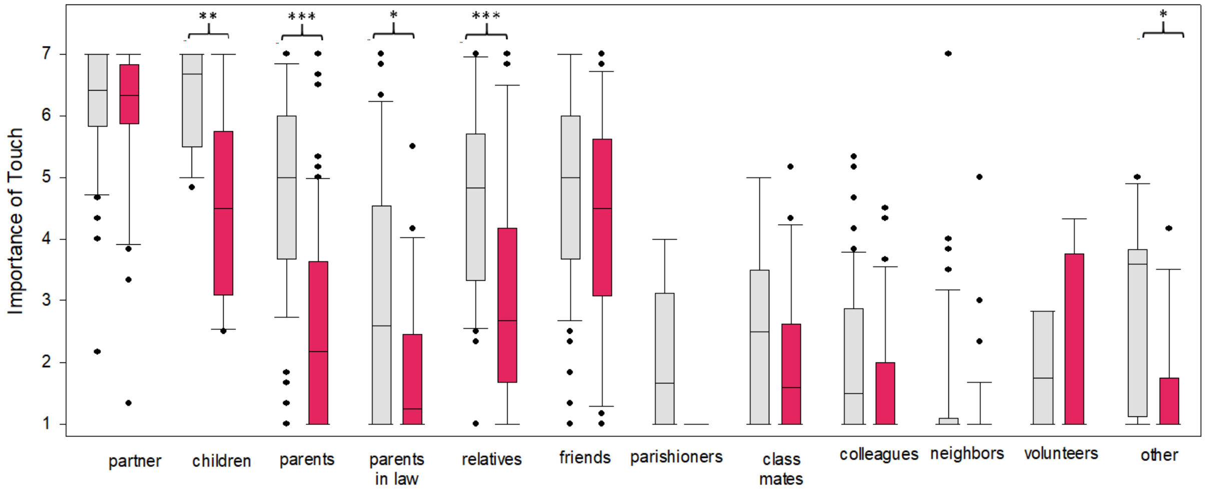

Figure S4. Importance of touch in different Social Domains.

Note. Comparisons include different sample sizes and statistical analyses have to be interpreted with care due to the restricted sample size for some comparisons.

Table S10

Results of the Multivariate Regression Analysis: CTQ as a predictor for the appraisal of touch

dependent variable	*β*	*SE*	*t*	*p*
need	-0.18	0.19	-0.96	.343
liking	-0.13	0.16	-0.81	.423
importance	< -0.01	0.15	-0.06	.955

Note. Four participants were excluded as outliers from these analyses.

Table S11

Results of the Multivariate Multiple Regression Analysis: The CTQ, capacity to feel close to others and their interaction as predictors for the appraisal of touch

	need				liking				importance			
	β	*SE*	*t*	*p*	*β*	*SE*	*t*	*p*	*β*	*SE*	*t*	*p*
CTQ	-0.02	0.17	-0.12	.907	-0.04	0.15	-0.25	.804	0.08	0.15	0.50	.618
att. clo.	0.75	0.19	4.03	< .001	050	0.17	3.00	.004	0.24	0.17	1.44	.156
CTQ*att. clo.	0.08	0.20	0.38	.702	0.14	0.18	0.76	.452	-0.13	0.18	-0.74	.461

Note. CTQ = childhood trauma questionnaire; att. clo. = attachment closeness. Four participants were excluded as outliers.

References

1. Walker EA, Unutzer J, Rutter C, Gelfand A, Saunders K, VonKorff M, et al. Costs of health care use by women HMO members with a history of childhood abuse and neglect. Archives of General Psychiatry. 1999;56(7):609-13.
2. Wolf M, Limberger MF, Kleindienst N, Stieglitz RD, Domsalla M, Philipsen A, et al. [Short version of the borderline symptom list (BSL-23): development and psychometric evaluation]. Psychother Psychosom Med Psychol. 2009;59(8):321-4.
3. Morey LC. The Personality Assessment Inventory: Professional manual. Odessa, FL: Psychological Assessment Resources. 1991.
4. Groves JA, Engel RR. The German Adaptation and Standardization of the Personality Assessment Inventory (PAI). J Pers Assess. 2007;88(1):49-56.
5. Beck AT, Steer RA, Brown G. Beck Depression Inventory-II: APA PsycTests; 1996.
6. Hautzinger M, Keller F, Kühner C. BDI-II Beck Depressions-Inventar. Frankfurt am Main: Harcourt Test Services; 2006.
7. Bernstein DP, Stein JA, Newcomb MD, Walker E, Pogge D, Ahluvalia T, et al. Development and validation of a brief screening version of the Childhood Trauma Questionnaire. Child abuse & neglect. 2003;27(2):169-90.
8. Klinitzke G, Romppel M, Hauser W, Brahler E, Glaesmer H. [The German Version of the Childhood Trauma Questionnaire (CTQ): psychometric characteristics in a representative sample of the general population]. Psychother Psychosom Med Psychol. 2012;62(2):47-51.
